# Experimental Models of Sarcopenia: Bridging Molecular Mechanism and Therapeutic Strategy

**DOI:** 10.3390/cells9061385

**Published:** 2020-06-02

**Authors:** Sakulrat Mankhong, Sujin Kim, Sohee Moon, Hyo-Bum Kwak, Dong-Ho Park, Ju-Hee Kang

**Affiliations:** 1 Department of Pharmacology, Hypoxia-related Disease Research Center, College of Medicine, Inha University, Incheon 22212, Korea; sakulratkulrat@gmail.com (S.M.); sujin2419@hanmail.net (S.K.); moon219@inha.ac.kr (S.M.); 2 Department of Kinesiology, Inha University, Incheon 22212, Korea; kwakhb@inha.ac.kr (H.-B.K.); dparkosu@inha.ac.kr (D.-H.P.); 3 Institute of Sports & Arts Convergence (ISAC), Inha University, Incheon 22212, Korea

**Keywords:** sarcopenia, aging, skeletal muscle, cellular senescence, experimental model

## Abstract

Sarcopenia has been defined as a progressive decline of skeletal muscle mass, strength, and functions in elderly people. It is accompanied by physical frailty, functional disability, falls, hospitalization, and mortality, and is becoming a major geriatric disorder owing to the increasing life expectancy and growing older population worldwide. Experimental models are critical to understand the pathophysiology of sarcopenia and develop therapeutic strategies. Although its etiologies remain to be further elucidated, several mechanisms of sarcopenia have been identified, including cellular senescence, proteostasis imbalance, oxidative stress, and “inflammaging.” In this article, we address three main aspects. First, we describe the fundamental aging mechanisms. Next, we discuss both in vitro and in vivo experimental models based on molecular mechanisms that have the potential to elucidate the biochemical processes integral to sarcopenia. The use of appropriate models to reflect sarcopenia and/or its underlying pathways will enable researchers to understand sarcopenia and develop novel therapeutic strategies for sarcopenia. Lastly, we discuss the possible molecular targets and the current status of drug candidates for sarcopenia treatment. In conclusion, the development of experimental models for sarcopenia is essential to discover molecular targets that are valuable as biochemical biomarkers and/or therapeutic targets for sarcopenia.

## 1. Introduction

Approximately three decades ago, sarcopenia was defined as a progressive decline of skeletal muscle mass, strength, and function [1]. In 2018, the European Working Group on Sarcopenia in Older People revised the definition of sarcopenia. In the revised version, low muscle strength, which is the most reliable measure of muscle function, became a primary parameter of sarcopenia [2]. It is widely accepted that aging is accompanied by the progressive decline of skeletal muscle mass, strength, and functions, which may be accelerated in some elderly people due to genetic, lifestyle, and environmental factors [3]. The etiologies of sarcopenia are not fully understood because the molecular mechanisms of these phenomena are complex and interrelated [4]. Multiple factors of sarcopenia have been identified, including cellular senescence, oxidative stress, mitochondrial dysfunction, fat accumulation, low-grade inflammation, inadequate nutrition, hormonal changes as well as a reduction in the number and regenerating capacity of satellite cells [4]. As there is no single key and inherent cause of sarcopenia, understanding the mechanisms of skeletal muscle aging might be essential for sarcopenia prevention and defining the therapeutic strategies of new drug discovery. Herein, we discuss the fundamental aging mechanisms leading to sarcopenia, summarize the in vitro and in vivo experimental models used to define the molecular and cellular changes in skeletal muscle aging, and provide potential strategies for prevention and treatment of sarcopenia as well as approaches for further development of anti-sarcopenia therapeutics.

## 2. Fundamental Aging Mechanisms that Contribute to Sarcopenia 

### 2.1. Cellular Senescence

**Figure 1:**
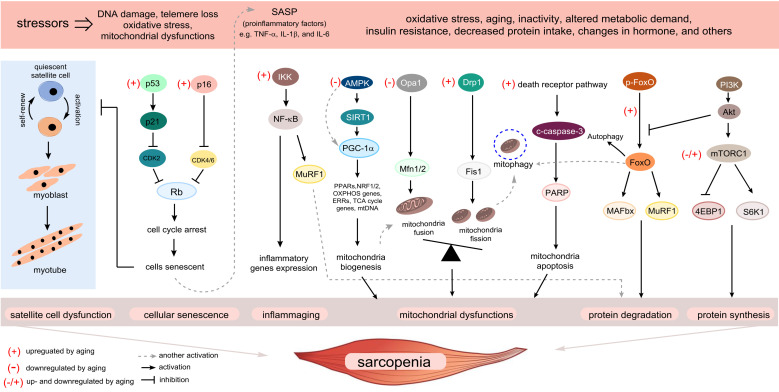
Overview of the fundamental aging mechanisms that contribute to sarcopenia. During the aging process, a variety of stressors engage various cellular signaling cascades, ultimately facilitating the progression of sarcopenia. Several senescence-inducing stimuli cause senescence of satellite cells leading to the loss of capacity to repair upon muscle injury. Many of these stimuli cause the upregulation of p53–p21^Cip1^ and p16^Ink4a^ pathways, which induce a temporal cell-cycle arrest by inhibiting cyclin-dependent kinase (CDK) 2 and CDK4/6, respectively. When cells enter a senescent state, the senescence-associated secretory phenotype (SASP) is expressed in those cells as a paracrine signaling pathway. “Inflammaging” and SASP production in senescent skeletal muscle cells converge on activation of nuclear factor kappa B (NF-κB) signaling, which induces upregulation of muscle ring finger 1 (MuRF1). Activation of the aging process causes mitochondrial dysfunction by deactivation of the sirtuin 1–peroxisome proliferator-activated receptor γ coactivator 1-α (PGC-1α) axis, which is primarily responsible for the maintenance of mitochondrial quality control. The accumulation of damaged mitochondrial DNA due to an imbalance in mitochondrial dynamics will be eliminated via mitophagy. However, under aging conditions, inefficient mitophagy eventually induces apoptosis. Aging interrupts the coordinated balance between protein synthesis and degradation, which activates and interconnects with multiple signaling pathways. Muscle atrophy F-box (MAFbx) and MuRF1, the major muscle-specific E3 ubiquitin ligases, are increased by activation of the transcription factor forkhead box O (FoxO) by aging stimuli, while the roles of Akt/mammalian target of the rapamycin (mTOR) signaling pathway in sarcopenia are controversial. Several possible mechanisms shown here could contribute to sarcopenia and be targets of intervention.

**Figure 2:**
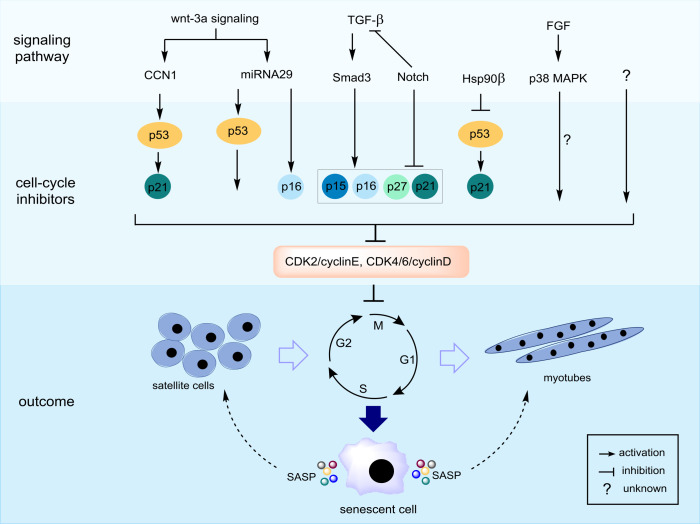
Possible molecular mechanisms of cellular senescence in skeletal muscle cells. The cell-cycle exit is mainly regulated by induction of cyclin-dependent kinase (CDK) inhibitors including p53–p21^Cip^, p16^Ink4a^/p15^Ink4b^, and p27^Kip1^. CCN1 and miRNA29 directly activate both p53 and p21^Cip^, while Hsp90β represses p53–p21^Cip^ signaling. Transforming growth factor (TGF)-β signaling activates CDK inhibitors via a pSmad3-dependent pathway. Notch and pSmad3 antagonize each other, and thus, Notch signaling inhibits these CDK inhibitors. Fibroblast growth factor receptor-p38 MAPK signaling has been identified as a critical pathway of deregulated cell-cycle progression and possibly influences cellular senescence; however, its downstream target(s) should be investigated. Skeletal muscle cells undergoing senescence show senescence-associated secretory phenotype (SASP), which can impinge on nearby cells.

Cellular senescence, which is characterized as a state of permanent cell-cycle arrest resulting from the finite replicative capacity of healthy cells, the so-called replicative senescence [5]. Cellular senescence is driven by a wide range of stressors, including DNA damage, shortening of telomere length, oxidative stress, mitochondrial dysfunction, oncogenic activation, and chemotherapeutic agents [6]. As depicted in Figure 1, there are several fundamental mechanisms of aging that contribute to sarcopenia. The stimuli induce cell-cycle arrest through various pathways, many of which activate two master regulatory axes, namely the p53–p21^Cip1^ and p16^Ink4a^-Rb pathways, and subsequently inhibit cyclin-dependent kinase (CDK) 2 and CDK4/6, respectively, resulting in hyperphosphorylation of retinoblastoma (Rb) protein, and eventually, cell-cycle exit [7]. In this review, we focus on the cellular senescence of satellite cells in the skeletal muscle, which might be associated with sarcopenia. Previous studies suggested that senescent satellite cells accumulated in skeletal muscles of aged rodents and elderly people by demonstrating the expression of p16^Ink4a^ and positive results of the senescence-associated β-galactosidase assay [8,9], although a recent study demonstrated no expression of p16 ^Ink4a^- and p21^cip1^-positive cells in skeletal muscle of elderly person [10]. The role of p16^Ink4a^ in sarcopenia has been investigated, particularly in satellite cells. Sousa-Victor et al. showed that the silencing of p16^Ink4a^ in geriatric satellite cells restored quiescence and muscle regenerative functions [8]. Baker et al. reported that the elimination of p16^Ink4a^ in the RubR1 progeroid mice delayed the development of sarcopenia [11]. On the other hand, inactivation of p19^Arf^ accelerated skeletal muscle deterioration [9]. The upregulation of skeletal muscle aging has not been widely studied and remains poorly understood; however, it may be induced through mechanisms associated with CCN1 (known as CYR61: cysteine-rich protein 61) [12], p38 mitogen-activated protein kinase (MAPK) [13], miRNA29 [14], Smad [15], and Hsp90β [16] (Figure 2). Indeed, the CCN1 and miRNA29 are regulated by Wnt-3a signaling, and both molecules contribute to muscle cell senescence by upregulation of p53 and p16^Ink4a^ [12,14]. Recently, the depletion of Hsp90β induced the upregulation of senescence markers via p53-dependent upregulation of p21^cip1^, which impaired muscle regeneration [16]. Notably, there are several molecular mechanisms and inducers of cellular senescence contributing to skeletal muscle aging, as described above. In addition, previous studies used various in vivo and in vitro experimental models, which showed inconsistent results. Therefore, the regulation of skeletal muscle aging needs to be explored rigorously. Although the elimination of senescent cells has been shown to prevent sarcopenia [11], injury-induced senescence is beneficial for skeletal muscle reprogramming in vivo [17]. Moreover, p21^Cip^ and p57^Kip2^ are essential to terminate the differentiation of muscle cells [18]. Interestingly, p53 delays functional decline of skeletal muscle cells in a p21^cip1^-dependent manner by inhibiting p16^Ink4a^ [19].

### 2.2. Senescence-Associated Secretory Phenotype (SASP) and “Inflammaging”

Previously, cellular senescence was well documented as a potent anticancer mechanism that prevents malignancies by limiting the replication of preneoplastic cells, but growing evidence has extended its known role to complex biological processes such as development, tissue repair, and aging-related diseases [6]. Accumulating data suggest that these complex biological processes are driven at least in part by the secretome of senescent cells (SASP), consisting of a range of cytokines, proteases, chemokines, and growth factors as well as extracellular vesicles. Importantly, the SASP can have either beneficial or detrimental effects depending on the specific composition of distinct cell types and different senescence-inducing stressors [20]. For example, oncogene-induced senescence produces SASP, which can, in turn, recruit the immune system for tumor clearance and eventual tumor regression [21]; however, SASP can also promote tumorigenesis [22]. 

Interestingly, aging-related senescent cells may be more persistent due to the deterioration of the immune system with aging [6]. Although the composition of SASP is not fully identified in several tissues including skeletal muscle, some inflammatory molecules in SASP are a major integral part of “inflammaging”. In this regard, the cellular senescence may mediate “inflammaging” through SASP production [23]. Accumulating evidence demonstrated that the serum levels of tumor necrosis factor (TNF)-α, interleukin (IL)-6, and C-reactive protein (CRP) are elevated in sarcopenia, typically up to 2–4-fold higher than those in young controls [24,25,26,27]. Therefore, accumulation of senescent cells may produce SASP, sustain “inflammaging”, which may underlie skeletal muscle aging and sarcopenia. Moreover, several community-based studies have shown an association between pro-inflammatory cytokines and sarcopenia. For instance, a cross-sectional study by Bain et al. revealed that the serum levels of IL-6 and TNF-α in an elderly sarcopenia group were higher than those in a non-sarcopenia group [26]. Recently, Marzetti et al. reported that higher levels of CRP, P-selectin, and interferon γ-induced protein 10 were found in people with physical frailty sarcopenia, whereas higher levels of myeloperoxidase, IL-8, monocyte chemoattractant protein 1, and platelet-derived growth factor BB were found in elderly people without frailty [25]. Recently, the combination of senolytic drugs consisting of dasatinib and quercetin selectively killed senescent cells in adipose tissue and endothelial or stem cells, respectively [28]. In addition, these combined senolytics reduced inflammatory activity in the adipose tissue and decreased SASP, which contributed to improvement in physical function and a healthy lifespan [29]. Thus, SASP and “inflammaging” could underlie skeletal muscle aging and mechanistic principles of developing a sarcopenic phenotype.

### 2.3. Progenitor or Satellite Cell Dysfunction

Skeletal muscles can regenerate over time by activation of skeletal muscle stem cells, such as satellite cells, to form new myofibers. In mature muscles, the satellite cells are present in a quiescent state in the absence of a stimulus, but they can be activated and induced to proliferate for damaged muscle replacement by extrinsic signaling [30]. Several studies reported that in advanced aging, the proliferative potential and number of skeletal muscle stem cells to repair injured muscle markedly decline, which is associated with sarcopenia [31,32]. The concept that satellite cell dysfunction could be one of the drivers of sarcopenia is derived from several observations [31,33,34]. For instance, the proportion and cross-sectional area (CSA) of type II fibers were found to be substantially reduced and these fibers had lower satellite cell content in elderly people than such fibers in young people [34]. Moreover, this concept was supported by the restoration of satellite cells that have therapeutic effects for sarcopenia [35,36,37]. However, the association of satellite cell reduction and dysfunction with sarcopenia is still controversial. There is evidence demonstrating that the reduction of satellite cells may lead to age-related muscle fibrosis but not directly accelerate sarcopenia [38] because depletion of satellite cells in adult mice does not affect muscle atrophy [38,39]. These studies suggested that satellite cells only play a crucial role in tissue regeneration and muscle mass maintenance following muscle injury, rather than size maintenance of unstimulated aged muscle fiber [38,40]. Nevertheless, we cannot exclude the roles of the loss of satellite cells and their functions in sarcopenia progression, at least with regard to impaired muscle fiber regeneration. In addition, extrinsic factors from satellite cell niches such as fibroblast growth factor [41], transforming growth factor-beta 1 (TGF-β1) [42], and myostatin [43], which regulate the intrinsic regenerative capacity of satellite cells, would be altered with aging processes and ultimately prevent efficient regeneration [44]. Moreover, parabiosis experiments indicated that impaired muscle regeneration in an aged rodent model was reversed by exposure to young circulation [44,45]. Therefore, the interplay between extrinsic and intrinsic factors, and possible mechanisms involved in sarcopenia should be further elucidated, which might provide opportunities for novel therapeutic interventions.

### 2.4. Oxidative Stress and Mitochondrial Dysfunction

With skeletal muscle aging, the maintenance of redox homeostasis balance declines, which leads to progressive oxidation of cellular constituents, including protein oxidation, lipid peroxidation, and oxidative DNA damage [46]. It is widely accepted that mitochondria serve as a significant source of oxidants as well as a primary target of oxidative stress. Mitochondrial damage accelerates the accumulation of reactive oxygen species (ROS) and cellular energy deficiency, particularly in the skeletal muscle, which may contribute to a complex sarcopenic phenotype [46,47]. The evidence showed that impaired mitochondrial respiratory capacity and increased ROS emission with aging is largely dependent on the individual physical activity, while pro-apoptotic factors released from mitochondria are commonly observed in cardiac and skeletal muscle with aging [48]. In other words, mitochondrial dysfunction and impaired quality control system with aging may increase the susceptibility of skeletal muscle to apoptotic loss of muscle cells. Interestingly, Picca A. et al., reported that the quantity of mitochondrial component was lower in secreted extracellular vesicles (EVs) of sarcopenic elderly people than that of non-sarcopenic elderly controls. However, the level of EVs in serum in sarcopenic patients was higher than non-sarcopenic controls [49], suggesting that the mitochondrial quality control through the secretion of EVs may be impaired in sarcopenic patients. Therefore, the mitochondrial components in EV may be used as a novel biomarker and therapeutic target of sarcopenia. In addition, mitochondria-derived EVs contain danger signals or molecules which activate sterile inflammatory pathways including Toll-like receptor and family pyrin domain-containing 3 (NLRP3) inflammasome. Mitochondrial dysfunction in skeletal muscle with aging may drive “inflammaging” that contributes to the development of sarcopenia [50].

Compared with young cells, senescent cells demonstrate upregulated mitochondrial oxidative metabolism to support the metabolic demand; however, such increased mitochondrial oxidation in senescent cells shows reduced redox balance and increased oxidative stress [51]. Oxidative stress causes mitochondrial dysfunction and cellular senescence, which are important contributors to aging-related diseases. The relationship between mitochondrial oxidative stress and cellular senescence has been explored in the past decades, but it is still not completely clear [52]. The premature-aging mice model showed sarcopenia phenotypes and presented a defect in mitochondrial DNA, mitochondrial biogenesis, and mitochondrial dynamics (fission and fusion) [53,54]. In addition, aged mice lacking superoxide anion scavenger (*Sod*1^−/−^) showed mitochondrial hydroperoxide generation and dysregulation of excitation-contraction coupling which contributes to muscle atrophy [55,56]. Indeed, several studies provided evidence that failure in mitochondrial dynamics leads to a negative consequence for the maintenance of muscle mass and function [54,57,58]. For example, dynamin-related protein 1, an essential cytosolic GTPase in the process of mitochondrial fission, plays roles in the regulation of Ca^2+^ homeostasis and controls skeletal muscle mass [57]. In addition, skeletal muscles of sedentary elderly subjects displayed decreased levels of optic atrophy 1 (OPA1), a crucial mitochondrial fusion protein, which is associated with skeletal muscle loss [58]. Therefore, in aging populations, many clinical observations supported that dysregulation of mitochondrial dynamics and function was associated with aging-induced skeletal muscle atrophy [59]. The correlation of mitochondrial dysfunction and muscle atrophy in aging has been explained in these studies and contributes to a better understanding of sarcopenia pathogenesis.

### 2.5. Protein Synthesis and Degradation

Skeletal muscle atrophy results from protein turnover under disrupted protein homeostasis conditions, i.e., abnormality in protein synthesis or protein degradation, which is controlled by multiple signaling pathways [60]. A major regulatory pathway of intracellular protein homeostasis is the Akt/mammalian target of the rapamycin (mTOR) pathway that has become an attractive target for sarcopenia research [61,62]. Indeed, activation of Akt signaling increases protein synthesis through activation of mTOR complex 1 (mTORC1)-mediated phosphorylation of S6 kinase (S6K) and inhibition of 4E-BP1. On the other hand, Akt can limit abnormal protein degradation in skeletal muscles and atrophy via phosphorylation of the transcription factor forkhead box O (FoxO) [62]. Although Akt/mTOR signaling pathways in muscles and other tissues are well understood, the implication of Akt/mTOR signaling during skeletal muscle aging is still under debate. Indeed, hyperactivation of mTORC1 signaling and decreased autophagy were found in aged muscles involved in sarcopenia, which were reversed by a low dose of an mTORC1 inhibitor [63,64]. However, the reason for mTORC1 hyperactivation in aged skeletal muscles is unclear. Rather, activation of 5′-adenosine monophosphate-activated protein kinase (AMPK) and increased autophagy induced by an mTORC1 inhibitor increased muscle mass and quality in a muscle-type dependent manner. Therefore, the effects of mTORC1 on sarcopenia might be tightly regulated and should be further investigated. The ubiquitin–proteasome system (UPS) and the autophagy–lysosome system are two types of proteolytic machinery that play a role in skeletal muscle homeostasis [59,60]. The UPS tightly regulates protein homeostasis via conjugation of multiple ubiquitin moieties (ubiquitination) mediated by ubiquitin ligases and degradation of ubiquitin-tagged proteins mediated by the proteasome complex. In skeletal muscles, the major muscle-specific E3 ubiquitin ligases include muscle atrophy F-box (MAFbx)/atrogin-1 and muscle ring finger 1 (MuRF1) [62,65]. It has been hypothesized that increased protein degradation via these types of machinery contributes to sarcopenia. Several pieces of evidence support this hypothesis, including the finding that expression levels of MAFbx and MuRF1 increase in skeletal muscles with aging progression and atrophy [65]. However, in another sarcopenic model, the skeletal muscle in rats with high-fat-diet-induced muscle atrophy did not show significantly elevated levels of MAFbx and MuRF1 [66]. In addition to the UPS-mediated protein degradation, an alternative proteolytic mechanism, autophagy, has been evaluated in the context of sarcopenia [67,68]. Aged cells demonstrate an insufficient clearance of accumulated intracellular waste materials because of a decline in the function of the autophagy–lysosome system during the aging process [69]. In addition, it has been suggested that a dysregulation in the elimination of damaged mitochondria through the autophagy machinery, a process termed mitophagy, can drive sarcopenia [70]. The autophagy flux is critical to preserve muscle mass and myofiber integrity [71]. Moreover, activation of autophagy facilitates muscle regeneration in sarcopenia [72]. However, it should be noted that the contradictory results may be influenced by the difference in experimental models (e.g., aging- versus high-fat-diet-induced sarcopenia), which have different protein turnover and autophagic responses; thus, the results should be interpreted with care.

## 3. Models for Studying Sarcopenia

Recently, research on sarcopenia has rapidly expanded and a variety of experimental models for the aging of skeletal muscles have been introduced. The establishment of an experimental model is a vital step in developing preventive and therapeutic strategies, and also in understanding the molecular pathways of sarcopenia [73,74,75]. As expected, aged animals are the most reliable and robust experimental models because they display the natural aging processes and may manifest factors that contribute to sarcopenia. In addition, it is anticipated that aged animals present symptoms, outcomes, or molecular mechanisms similar to those found in human sarcopenia patients [73]. However, several limitations exist regarding the use of aged animals, including but not limited to time-consuming experiments, high cost, and variation in conditioned phenotypes. Thus, researchers have made an effort to develop appropriate animal models to recapitulate human sarcopenia. Regarding cell line models, a comprehensive understanding of the pathophysiology of sarcopenia is challenging to undertake due to difficulty in finding an appropriate condition to stimulate the aging of skeletal muscle cells, induce atrophy, and mimic senescence niches [76]. To overcome these limitations, primary skeletal muscle cell culture and in vivo systems have been studied. We summarize the reliable approaches for the investigation of sarcopenia in both in vivo and in vitro models.

### 3.1. In Vitro Models

Several skeletal muscle cell culture models have been used for investigating the pathophysiology of sarcopenia. The most commonly used in vitro models to study the molecular aspects of skeletal muscle cells are the rat skeletal muscle L6 cells and murine C2C12 cells [77,78,79]. Although the in vitro models using immortalized cell lines for skeletal muscle wasting are not sufficient to understand the mechanisms of sarcopenia, they have been used to mimic sarcopenia in vivo in the aspect of a few variations in the protein, transcriptional, and molecular mechanisms involved in sarcopenia development [76]. Human primary skeletal muscle cells have been used in recent years to study myogenesis and muscle aging [80,81]. Compared with the use of immortalized cell lines, in vitro culture models using primary muscle cells from humans or animals with wide ranges of age might have advantages for understanding the mechanisms of sarcopenia. The induction of immortalized skeletal muscle cell lines to imitate pathophysiology of sarcopenia by using several substances including oxidative stress (H_2_O_2_), sphingophospholipid (ceramide or palmitate), inflammatory cytokines (TNF-α) as well as dexamethasone are discussed in the present review (Table 1).

#### 3.1.1. H_2_O_2_

It is widely accepted that in sarcopenia, the level of oxidative stress is increased and ROSs are accumulated in skeletal muscles [82]. H_2_O_2_ is known as a nonradical ROS that can diffuse across cellular membranes and increase the intracellular ROS levels, especially in skeletal muscles [83]. H_2_O_2_-stimulated skeletal muscle cells can induce several mechanisms involved in the pathogenesis of sarcopenic muscle characteristics. H_2_O_2_ has been used to stimulate muscle cells as a model for investigating oxidative stress-induced damage in both myoblasts and differentiated myotubes [84]. Treatment of skeletal muscle cells with H_2_O_2_ at various concentrations (10 μM−4 mM) and durations induces cellular damage, including oxidative stress [85], apoptosis [86,87,88], endoplasmic reticulum stress [89], autophagy [87,90,91], and mitochondrial dysfunction [92,93]. Indeed, oxidative stress-induced apoptosis has been reported on the exposure of myotubes to H_2_O_2_, wherein millimolar concentration (1–4 mM) of H_2_O_2_ induced an increase in apoptotic DNA fragmentation, increase in Bax level, decrease in Bcl-2 level, and activation of caspase-3 in a time-dependent manner (24–96 h) [86]. However, although L6 myoblasts are considered to be more sensitive to the cytotoxic effect of H_2_O_2_ than C2C12 myoblasts [88], a study showed that 200 μM of H_2_O_2_ for 24 h was sufficient to stimulate apoptosis in C2C12 myoblasts [87]. Meanwhile, exogenous H_2_O_2_ also activates peroxisome proliferator-activated receptor γ coactivator 1-α (PGC-1α)/AMPK expression in C2C12 myoblasts; this signaling plays a central role in the adaptation of cellular energy metabolism, mitochondrial biogenesis, and antioxidant defense [93]. In addition, H_2_O_2_-treated skeletal muscle cells have been used to study the role of peroxisome proliferator-activated receptor-γ (PPAR-γ) in acute exercise-induced oxidative stress in vivo [94]. H_2_O_2_-mediated oxidative stress in skeletal muscle cells is suitable for mimicking the role of oxidative stress in sarcopenia pathogenesis. However, this model still lacks other characteristics of sarcopenia pathogenesis such as cellular senescence and secretion of “inflammaging” mediators. Hence, whether H_2_O_2_ can stimulate other aging mechanisms should be investigated.

#### 3.1.2. Ceramide and Palmitate

Sphingolipids have been defined as a class of bioactive lipids, such as ceramide, sphingosine, and ceramide-1-phosphates, that are capable of regulating fundamental biological processes in skeletal muscles [95,96]. Among these, ceramide is a potent bioactive sphingolipid, which is accumulated in skeletal muscles by either activation of sphingomyelinase or via de novo synthesis from palmitate [95]. Ceramide has been reported to inhibit skeletal muscle cell (L6) myogenesis because inhibition of its de novo synthesis enhances myoblast differentiation [97]. Russ et al. indicated that ceramide was increased in aged rats in association with increased autophagy and age-related sarcoplasmic reticulum stress [69,98]. Ceramide is also increased in skeletal muscles of obese insulin-resistant humans [99], and such an increase accompanies obesity-related sarcopenia [100]. It is well established that ceramide can limit insulin-mediated Akt/protein kinase B (PKB) signaling and also directly activate a form of protein kinase C zeta (PKCζ), a negative regulator of Akt signaling [96]. Therefore, the negative roles of ceramide in the development of insulin resistance, which contributes to sarcopenia pathophysiology, have been studied. In fact, some studies showed that C-2 ceramide promoted senescence of muscle cells [78,101], increased the level of oxidative stress, and induced mitochondrial fission [102]. It has been reported that palmitate-treated muscle cells mediated sphingolipid synthesis, particularly that of ceramide, which is a primary modulator of metabolism and insulin resistance [103,104]. Moreover, palmitate delayed myoblast differentiation, induced insulin resistance, induced cellular senescence [78,101,105], impaired autophagic flux [101], and increased atrogin-1 and MuRF1 gene expression [105]. Ceramide- and palmitate-treated muscle cells might provide a suitable sarcopenia model, at least concerning skeletal muscle senescence and insulin resistance associated with sarcopenic obesity.

#### 3.1.3. Inflammatory Cytokines

Low-grade inflammation is highly prevalent in the elderly and can cause aging-related loss of skeletal muscles [24]. Pro-inflammatory cytokines, especially TNF-α, have been implicated as stimulators of muscle catabolism [106,107]. Low concentrations of TNF-α (3–100 ng/mL) can stimulate muscle atrophy [108,109,110,111]. TNF-α signaling can alter protein degradation by directly activating the UPS in rat soleus muscles [112] and trigger the death receptor-mediated pathway [113]. Accumulating evidence indicates that the correlation between the levels of muscle-specific ubiquitin ligases, MAFbx and MuRF1, and TNF-α becomes stronger with the aging-related loss of skeletal muscles [106,109]. In addition, TNF-α induced MAFbx gene expression via the p38 MAPK pathway in C2C12 and L6 skeletal muscle cells [109,111]. Meanwhile, TNF-α decreased the expression of myogenesis factors and the diameter of myotubes in a dose-dependent manner through various signal transduction pathways, including PI3K/PKB, MAPK, and nuclear factor kappa B (NF-κB) signaling pathway, in L6 cells [111].

Moreover, TNF-α-treated muscle cells have been used to study therapeutic interventions for muscle wasting diseases [110,116]. Wang et al. demonstrated that TNF-α induced myotube atrophy in C2C12 cells by regulating the Akt/mTOR and FoxO1/3a signaling pathways, which was prevented by resveratrol [110]. Interestingly, TNF-α was able to induce de novo ceramide synthesis in L6 cells but not in C2C12 cells. As mentioned in Section 3.1.2, it is well established that ceramide plays a detrimental role in skeletal muscles [124]. TNF-α-induced skeletal muscle cells are a useful in vitro model because TNF-α levels are increased during aging and associated with sarcopenia. Nevertheless, more experimental evidence is needed to understand the effects of TNF-α on other aging mechanisms such as cellular senescence and mitochondrial dysfunction.

#### 3.1.4. Glucocorticoids (GCs) and Dexamethasone

GCs are widely used as anti-inflammatory and immunosuppressive agents. However, the excessive doses and prolonged use of GCs contribute to several adverse side effects, including skeletal muscle wasting and weakening [125]. Dexamethasone is a synthetic GC and it has been used to induce skeletal muscle weakness in animal experiments [126,127]. Moreover, dexamethasone blocked protein synthesis signaling through the Akt/mTOR pathway and mediated response through the GC response element (GRE) [125]. In addition, dexamethasone-treated muscle cells are commonly used to study amyotrophy. Dexamethasone has been demonstrated to upregulate the ubiquitin–proteasome machinery through increased MAFbx and MuRF1 expression [119,128]. Exposure of C2C12 myotubes to dexamethasone robustly increased MAFbx, MuRF1, and myostatin expression [119,123], reduced diameter of myotubes and decreased MyoD and myogenin expression [123]; similar results were observed in L6 cells [122]. Dexamethasone regulates these outcomes through various mechanisms, including PKA/B and Akt pathways [129], NAD+-dependent protein deacetylase sirtuin 1 (Sirt1)/PGC-1α involved in mitochondrial biogenesis, and FoxO1/3a transcription factors [119]. However, the pathophysiology of sarcopenia comprises not only an imbalance between protein anabolic and catabolic processes but also sophisticated aging processes [4,130]. Limited data exist regarding whether other aging-related mechanisms such as cellular senescence and “inflammaging” are activated by dexamethasone. Therefore, dexamethasone-treated muscle cells do not exemplify the natural aging of skeletal muscles. 

#### 3.1.5. Primary Skeletal Muscle Cells and Single Myofiber

Several studies regarding skeletal muscles have been performed using immortalized muscle cell lines such as C2C12 and L6 cells. Although these cell lines have been used for delineating several regulatory mechanisms of skeletal muscles, inconsistent results have been obtained with these cell lines depending on the origin and cell conditions. Primary myoblasts closely resemble their parental tissue and may mimic more closely an in vivo condition than immortalized cell lines [131]. Primary skeletal muscle cells have been used for examining the regeneration capacity of satellite cells and developing sarcopenia therapeutics [132,133,134]. In particular, the aging muscle fiber loss in rodents may not be similar to that in humans [134]. For example, silencing of upregulated p16 in geriatric mice restores the proliferative activity of satellite cells, however, a recent publication reported that the upregulation of p16 in human skeletal muscle tissue was not observed [8,10]. Therefore, primary myoblasts derived from human skeletal muscle biopsy are considered a valuable tool for studying skeletal muscle physiology and pathophysiology [80]. Human primary skeletal muscle cells can be indicative of the natural environment and genetic background as well as the disease circumstances in vitro [81]. These cells retain at least some of the characteristics of their donors. For instance, human primary myoblasts from non-diabetic but insulin-resistant patients showed a defect in insulin signaling [135]. Moreover, myotubes derived from severely obese with type 2 diabetes had higher lipid accumulation capacity and lower lipolysis rate as compared to obese patients without type 2 diabetes [136]. The properties of human primary skeletal muscle cells might follow the characteristics of donors including age, genetic background and disease status. In addition, Brzeszczyńska et al. used human primary skeletal muscle cells to demonstrate that healthy young people have more muscle regeneration capacity than older people with sarcopenia [137]. This finding enabled the use of human primary skeletal muscle cells for studying the mechanisms underlying sarcopenia. Moreover, several research groups developed novel techniques to isolate and obtain these cells [80,138,139]. However, the use of these cells is challenging, and there is also a lack of consensus concerning the different procedures and culture conditions. Therefore, standardization of the methods and culture conditions for human primary skeletal muscle cells is necessary.

In addition, ex vivo single myofiber is one of the powerful tools for skeletal muscle research. Ex vivo single myofiber from biopsied samples of murine or human is beyond the traditional cell culture (in vitro) systems which maintain some of the in vivo circumstances. Even though the preparations of ex vivo single myofiber are delicate and technically challenging, intact myofibers show effective attraction [140]. Moreover, the ex vivo single muscle fiber offers the method to evaluate muscle type-specific manner. For example, Murgia M. et al. analyzed the single muscle fiber proteomics of human muscle and revealed that the carbohydrate metabolism in elderly decreased in fast- but increased in slow-twitched muscle fiber. While oxidative phosphorylation decreased in both types of muscle [141]. Of note, the single myofiber approach is technically challenging due to the difficulty of the procedure, low yield of fiber during the isolation process, isolation without fiber damaged, as well as a specific protocol of each muscle type [140,142]. However, ex vivo analysis of the single myofiber is appreciated as a good approach, which enables one to investigate the effect of sarcopenia interventions at the level of specific myofiber types in feature of contractile property and metabolisms. 

### 3.2. Animal Models

Animal models are indispensable for investigating the molecular mechanisms and developing novel therapies or molecular diagnostic tools. The use of aged animals has several advantages over inducing muscle atrophy, and it is frequently used to study therapeutic interventions for sarcopenia. Furthermore, one of the most important advantages is that a therapeutic effect or a mechanism should be necessarily validated in normal aged mammals to exclude that sarcopenia is specific of the genetic models. However, the use of aged animals is not only costly but also time-consuming (requires >20–24 months). Thus, mouse strains with accelerated aging and genetically modified mice have become prevalent as models to investigate skeletal muscle wasting [66,76,143]. Herein, we discuss animal models that have been used to study sarcopenia based on the molecular mechanisms of each model (Table 2).

#### 3.2.1. Aged Animals

Among the in vivo models used to explore sarcopenia, the use of aged animals provides various advantages mainly because comorbidities similar to those found in humans with sarcopenia are expected to occur in aged animals. The most frequently used animals for investigating sarcopenia are male Sprague-Dawley rats because they are less susceptible to sarcopenia than female rats. A significantly lower muscle CSA at 16 months of age was observed in male Sprague-Dawley rats, but not in female rats [66]. Likewise, at 18 months of age, the gastrocnemius muscle mass was decreased and progressively atrophied in male rats [64]. In addition, a reduction in quadriceps muscle CSA was observed in 20- and 23-month-old male Wistar rats [144]. The CSA of myocytes was also dramatically reduced in 20- and 24-month-old C57BL/6J mice when compared with that in 6-month-old mice [47]. Furthermore, aged animals fed with a high-fat diet can be used to study sarcopenia because fat consumption can exemplify a constant risk factor for developing obesity and age-related muscle wasting [66,144]. However, it should be noted that the end point of experimental outcomes might be affected by the variation in food intake and diet composition. Several molecular signaling mechanisms associated with progressive sarcopenia, such as oxidative stress, mitochondrial function (PGC-1α and Sirt1), and protein synthesis and proteolytic systems (MAFbx/MuRF1 and Akt/S6K/4E-BP1) signaling, have been investigated using aged-animal models [47,66,144]. Moreover, aged-animal models enable researchers to conduct translational research and obtain a better understanding of the sarcopenic phenotype. Recently, based on the findings that longevity-associated variant (LAV) haplotypes associate with frailty in elderly people, *LAV-BPIFB4* gene therapy delayed frailty progression in aged mice [153]. Despite the advantages, animal models with natural aging are time-consuming and not highly cost-effective. Therefore, animal strains with accelerated aging are preferred when considering the time duration.

#### 3.2.2. Senescence-Accelerated Mouse (SAM)

The development of SAM strains has been valuable for aging research. SAM strains are a series of inbred mouse strains that exhibit accelerated senescence and short lifespan. The characteristics of senescence-prone mouse (SAMP) strains are similar to symptoms observed in elderly people [154]. For example, SAMP8 showed the neurodegeneration symptoms and pathologies of Alzheimer’s disease. Many aging-related diseases have been well characterized using SAMP strains based on the specific phenotypes, and senescence-resistant mouse (SAMR) strains have been used as controls [155]. Although the primarily established SAMP strains have no phenotypes that are associated with muscle atrophy [155], the up-to-date SAMP strains are frequently used as a model in sarcopenia research, particularly SAMP8. It has been reported that SAMP6 mice at 60 weeks of age showed a decrease in the size of tibialis anterior muscle fibers [156]. SAMP10 mice showed aging-induced skeletal muscle wasting, and this strain has been used to investigate the effect of exercise training on sarcopenia [148]. Among SAMP strains, SAMP8 showed the greatest deterioration of skeletal muscle mass and contractility compared with SAMR1 control [75,157]. SAMP8 mice at 8 months of age are considered to be at a presarcopenia stage, while those at 10 months of age might be considered to be at a sarcopenia stage [145]. Guo et al. showed that the number of type II muscle fibers, which are predominant in gastrocnemius muscles, reached a peak at 7 months of age and then declined gradually in SAMP8 mice [145]. Consequently, SAMP8 mice have been used to investigate novel sarcopenia therapeutics and assess their efficacy [120,146,147]. For example, gosha-jinki-gan alleviated skeletal muscle atrophy through Akt/FoxO4/MuRF1- and AMPK/PGC-1α-associated mitochondrial dysfunction in SAMP8 mice [146]. Furthermore, high-fat-diet-fed SAMP8 mice exhibited susceptibility to aging-related muscle wasting, and the muscle weight was decreased by 15.3% at 6 months of age compared with that at 2 months of age [147]. Several observations suggested that the use of SAMP strains is cost-effective for sarcopenia research. Although SAMP strains are a valuable tool for sarcopenia research because of the shorter time required to reach senescence, they may not always show the characteristics of natural aging. Thus, cautious interpretation of the results of such research is necessary.

#### 3.2.3. Genetically Engineered Animal Models

The use of genetically engineered mice in sarcopenia research has progressively increased over the past years and has contributed to the knowledge of this geriatric disease. Decreased muscle protein synthesis and increased muscle protein degradation have been proposed to underlie the pathogenesis of sarcopenia [62]. Several studies on skeletal muscle atrophy have used mice with genetic modifications. For example, the Eif4ebp1^−/−^ and Eif4ebp2^−/−^ double knockdown (4EBP1/2 DKO) mice have been used to study the regulation of skeletal muscle protein synthesis. The depletion of 4E-BPs is associated with perturbed energy metabolism in skeletal muscles, and it has also been suggested that 4E-BP can be a target of sarcopenia intervention [158]. The use of growth hormone receptor knockout (GHR^−/−^) and bovine GH mice provided evidence that the insulin-like growth factor 1 (IGF-1) signaling pathway predominantly regulates myostatin, which is a negative regulator of myogenesis [159]. The correlation between mitochondria and muscle atrophy has been elucidated using Opa1^−/−^ mice. OPA1 is a mitochondrial fusion protein, which can control muscle proteolysis/protein synthesis [58,160]. Noteworthy, lacking CuZn superoxide dismutase (*Sod1*^−/−^) mice elevated mitochondria hydroperoxide generation, subsequently muscle atrophy. Indeed, *Sod1*^−/−^ has been suggested as a potential tool for studying the pathogenesis of sarcopenia and testing sarcopenia interventions. [55,161]. Furthermore, transgenic mice models can offer novel candidates for musculoskeletal modulators. For instance, Fam210a is a novel gene identified using a transgenic mice model that showed an association between skeletal muscle strength and bone structure. It might be a new target of sarcopenia and osteoporosis treatment [162]. Genetic engineered animal models can provide insights into various mechanisms underlying sarcopenia. However, studies involving these models should be interpreted with caution. These mice may show typical and/or exaggerated features of sarcopenia, and they often show appearance features not seen under normal aging conditions. Lastly, mouse genetic knockouts can examine only a few specific pathways at a time and provide a limited understanding of the disease.

#### 3.2.4. Hindlimb Suspension (Microgravity)

The hindlimb suspension (HLS) model has been widely used to mimic the disuse of skeletal muscle and microgravity conditions [73,149]. In this procedure, the hindlimb muscles are unloaded by attaching an orthopedic tape around the proximal two-thirds of the animal’s tail. The elevated animal’s hindlimbs are adjusted and suspended from the ground, while the forelimbs remain free [149]. HLS can contribute to muscle atrophy by disrupting several cellular processes such as inducing oxidative imbalance [149], mitochondrial dysfunction [163], autophagy [150], and improper protein synthesis/degradation [164]. Hindlimb unloading disrupts the redox balance by inducing oxidative stress and decreasing antioxidant production (e.g., catalase and glutathione peroxidase) [149,165]. In addition, Cannavino et al. suggested that HLS interrupted metabolic mechanisms, which resulted in muscle atrophy [163]. They showed that HLS induced mitochondrial dysfunction by decreasing mitochondrial fusion protein (Mfn1, Mfn2, and OPA1) expression, induced atrogene expression through AMPK/FoxO3 activation, and ultimately resulted in a loss of muscle mass [163]. The HLS model for studying sarcopenia is typically used in aged animals, which can show exacerbated muscle atrophy [150,151]. Two-week hindlimb unloading in both rats and mice showed a loss of myofibrillar proteins in soleus muscles [151,152,164]. The duration of suspension affects the degree of muscle mass loss. Rats subjected to short-term HLS showed alterations in metabolic mechanisms; these alterations occurred in soleus muscles rather than gastrocnemius muscles. The appearance of this phenotype can be regarded as reasonable because the type I (soleus) muscle is considered an aging-resistant “gravity muscle” while the type II muscle is considered an aging-susceptible fast-twitch muscle. In contrast, rats subjected to prolonged HLS (>7 days) showed a decrease in the CSA of type IIB fibers of gastrocnemius muscles [163,165]. The current evidence indicates that the HLS model is useful as a model of inactivity-related sarcopenia rather than sarcopenia associated with skeletal muscle aging. The use of old animals together with short-term HLS can probably be an effective model for sarcopenia combined with aging-related inactivity. However, it should be noted that the ethical concern may have some limitations to use the HLS model.

## 4. Molecular Strategies to Develop Therapeutics for Sarcopenia

Although the mechanisms that initiate sarcopenia are not fully understood, several molecular strategies for developing sarcopenia therapeutics have been suggested. The imbalance of protein homeostasis is considered an initial process that mediates the age-related loss of muscle mass. Muscle atrophy occurs when the muscle is disused, immobilized, and/or denervated, as well as during starvation, which reduces muscle protein synthesis [61,62]. IGF-1/PI3K-Akt signaling is primarily responsible for muscle protein synthesis [166]. Skeletal muscle hypertrophy is promoted by activation of mTOR and glycogen synthase kinase 3β, while Akt inhibits protein degradation through FoxO-mediated proteasome activity [166]. MAFbx and MuRF1 are downregulated by the transcription factor FoxO3A [60,62]. The promotion of skeletal muscle mass through IGF-1/Akt/mTOR signaling and suppression of protein degradation via inhibition of atrogenes, which may play a pivotal strategy for sarcopenia intervention. However, it has been demonstrated that activation of mTORC1 was detected in aged human muscle but did not induce protein synthesis [61,167]. Very recent evidence indicated that mTORC1 signaling is not essential for the maintenance of muscle size in adult sedentary mice, however, rapamycin treatment of adult mice does not appear to have a positive effect on muscle function [168]. In addition, partial inhibition of mTORC1 is helpful to delay the progression of sarcopenia [64]. Unlike Akt/mTOR signaling, AMPK regulated muscle fiber protein through inhibition of mTORC1. Moreover, AMPK can directly activate FoxO-dependent protein degradation [169]. mTORC1 is regulated by several upstream signaling pathways and has been implicated in several physiological conditions related to protein synthesis, autophagy, cell survival and energy production. Therefore, further investigations are essential to target mTOR signaling for sarcopenia therapeutics.

Another attractive possibility to attenuate the loss of skeletal muscle mass in aging is the PGC-1α/Sirt1 signaling pathway. It has been demonstrated that activation of PGC-1α can prevent muscle atrophy via suppression of FoxO3 and NF-κB [170]. Apart from its roles in the regulation of protein degradation, the PCG-1 family tightly regulates mitochondrial biogenesis and functions [171]. Exercise training increases levels of PCG-1 in skeletal muscles; therefore, the activation of PGC-1α can explain how exercise prevents muscle atrophy [172]. The activation of PCG-1α and its coactivators not only inhibits atrogenes but also compensates for the mitochondrial dysfunctions in sarcopenia. In addition, accumulation of oxidative stress and low-grade chronic inflammation accompanies sarcopenia and alters mitochondrial functions through several mechanisms [173]. The beneficial effects of antioxidants have been broadly examined to target oxidative damage and ROS generation related to sarcopenia. For instance, resveratrol showed therapeutic potential against skeletal muscle atrophy through the activation of the AMPK/Sirt1 pathway in an animal study [174]. Interestingly, a recent study showed that higher dietary intake of antioxidants including vitamin E, and vitamin C (especially, vitamin C) and carotenoid were reversely associated with indices of sarcopenia in women [175]. Consistency, carotenoid- and polyphenol-rich diet ameliorated the skeletal muscle atrophy [176]. However, the use of the antioxidant supplement for sarcopenia patients need to be further explored. In addition, therapies targeting of senescence in satellite cells can be another approach for sarcopenia therapeutics. Elimination of senescent satellite cells by senolytic drugs has been recently prevailing [177], which can provide both preventive and therapeutic strategies for sarcopenia. Senolytics drugs not only eliminate senescent satellite cells but also decrease the level of SASP, which subsequently recover the activity of remaining satellite cells for muscle regeneration. Nativoclax and FOXO4-DRI peptide that showed beneficial on muscle stem cells. Interestingly, FOXO4-D-Retro-Inverso (FOXO4-DRI) peptide showed improved physical performance in aged mice [178,179]. Most of the research for targeting cellular senescence in skeletal muscle has focused on satellite cells but lack of evaluation for the effects of the inhibition on frailty and physical performance. Therefore, the effect of senolytic drugs on skeletal muscle mass, strength and physical performance in an aged-animal and -human study is necessary for further investigation. Alternatively, inhibition of satellite cell senescence can be another target of a preventive strategy. Chronically elevated SASP and inflammatory mediators have been linked to the disruption of muscle homeostasis [180]. Thus, elimination of satellite cell senescence, elevation of antioxidant activity, increase in anti-inflammatory but decrease in pro-inflammatory activity or preservation of mitochondrial function could be considered as promising strategies for sarcopenia treatment.

Muscle loss can be induced by an increase in myostatin, which is a myokine involved in the negative regulation of myogenesis. Myostatin has been extensively studied as a molecular target of drug candidates for sarcopenia treatment [3,181]. It is a member of the TGF-β superfamily [182], and it binds to activin type II receptors on muscle fibers resulting in the activation of Smad signaling. This transduction signaling can also inactivate Akt signaling and its downstream effectors such as mTOR and FoxO [3,60]. Although the findings for whether myostatin levels increase with aging are inconsistent, positive effects of myostatin inhibition on muscle quality have been reported. Landogrozumab (LY2495655), an antimyostatin antibody, has been used in a phase II clinical trial of patients with sarcopenia and yielded positive results [183]. Bimagrumab, which is another drug candidate for sarcopenia therapeutics and targets activin type IIb receptors, was used in a phase II proof-of-concept study and led to increased and sustained thigh muscle volume [184]. These positive results provide new insights into sarcopenia intervention research; however, the development of novel molecular strategies and potential therapeutic approaches for sarcopenia is still necessary to deal with this complex geriatric disease.

## 5. Conclusions

Based on the molecular mechanisms of sarcopenia, several in vitro models have been suggested to study sarcopenia. However, the processes underlying sarcopenia development do not only include sarcopenic mechanisms but also include indirect mechanisms related to the skeletal muscle environment. Therefore, the use of in vitro models has inevitable limitations even though such models have several advantages. In particular, primary skeletal muscle cells from donors with different biological conditions (e.g., aging and insulin resistance) are the best in vitro models for studying sarcopenia or other forms of sarcopenia. In addition, the use of an appropriate in vitro model will be a good strategy to screen molecular targets or identify therapeutic compounds. Compared with in vitro models, the use of animal models with natural aging or genetically modified animals offers the advantage of studying the systemic effects of sarcopenia, organ-level molecular patterns, and biochemical biomarkers. However, the use of animals is time-consuming or has low cost-effectiveness. In addition, the use of experimental models for sarcopenia should be linked with translational research that is further progressed to clinical research. Furthermore, pre-clinical research in an aged-animal model is a critical platform that can provide reasonable evidence to progress in clinical researches as well as to achieve a translational value. Currently, several candidates for sarcopenia therapeutics have been introduced in clinical trials at different phases, although the molecular mechanisms of sarcopenia are yet to be fully elucidated. Therefore, the development of experimental models for sarcopenia is essential to identify molecular targets that are valuable biochemical biomarkers and/or therapeutic targets for sarcopenia.

## Figures and Tables

**Table 1:** In vitro models for mimicking sarcopenia based on aging-associated mechanisms.

Stimulator	Muscle Cell	Mechanism Signaling Involvement	Ref.
H_2_O_2_	Myoblast	↑DNA-damage ↑apoptosis ↑autophagy	[85,87,91,114]
	Myotube	↑apoptosis ↑autophagy ↑ubiquitin-proteasome system (atrogin1 and MuRF1) ↑ER stress ↓mitochondrial function ↓MHC type II	[86,88,89,90,92,93]
Ceramide & palmitate	Myoblast	↑autophagy ↑cellular senescence ↓myogenesis	[78,97,101,115]
	Myotube	↑autophagy ↑cellular senescence ↑mitochondrial fission ↑insulin resistance ↑ubiquitin-proteasome system (atrogin1 and MuRF1)	[101,102,104,105]
TNF-α	Myoblast	↑apoptosis ↓myogenesis	[116,117]
	Myotube	↑apoptosis and necrosis (high conc.) ↑ubiquitin-proteasome system (atrogin1 and MuRF1) ↓myogenesis	[109,110,111]
Dexamethasone	Myoblast	↓myogenesis	[118]
	Myotube	↑apoptosis ↑ubiquitin-proteasome system (atrogin1 and MuRF1) ↓autophagy ↓mitochondrial content and functions ↓protein synthesis ↓MHC type II ↓myotube diameter	[119,120,121,122,123]

↑; increase, ↓; decrease.

**Table 2:** Animal models for investigating sarcopenia.

Animal Models		Major Phenotypes	Molecular Mechanisms Associated Sarcopenia	Ref.
Aged Animals	Male Sprague-Dawley rats (16 months, +HFD)	↓muscle fiber CSA	↑caspase-3-dependent apoptosis ↔ Akt signaling (MAFbx and MuRF1)	[58]
	Male Sprague-Dawley rats (24 months)	↓muscle fiber CSA ↓muscle mass	↑MuRF-1 and atrogin1 ↑senescence (p21 and p16) ↑ mTOR signaling (p70S6K/4E-BP1)	[64]
	Male Wistar rats (20–23 months, +HFD)	↓muscle fiber CSA ↑muscular fat	↓protein synthesis signaling (mTOR/p70S6K/4E-BP1)	[144]
	C57BL/6J mice (12 and 24 months)	↓ muscle fiber CSA ↓ muscle mass	↑oxidative stress ↑mitochondrial dysfunction ↑MuRF-1 and atrogin1 ↓protein synthesis signaling (Akt/p70S6K/IGF-1)	[47]
Senescence-Accelerated Mouse (SAM)	SAMP8 (60 weeks)	↓ muscle fiber CSA ↓ muscle mass	No evidence	[75]
	SAMP8 (32 and 40 weeks)	↓ muscle mass ↓ muscle strength and function	No evidence	[145]
	SAMP8 (38 weeks)	↓ muscle fiber CSA	↑muscle atrophy (FoxO4/MuRF1, atrogin1) ↑mitochondria dysfunction (AMPK/PGC-1α signaling)	[146]
	SAMP8 (32, weeks, +HFD)	↓muscle mass	↓protein synthesis signaling (Akt/p70S6K) ↓insulin signaling	[147]
	SAMP10 (40 weeks)	↓number of muscle stem cells	↓protein synthesis signaling (mTOR/Akt/FoxO3) ↓mitochondria biogenesis (PGC-1α)	[148]
Knock-out (KO) mice	CuZn superoxide dismutase KO mice (*Sod1*^−/−^)	↓muscle mass ↓muscle strength	↑muscle atrophy ↑mitochondria hydroperoxide production	[55,56]
	Optic atrophy 1 KO mice(*Opa*^−/−^)	↑muscle loss and weakness ↑aging phenotype (white hair & kyphosis)	↑mitochondrial dysfunction ↑muscle atrophy ↓ myogenesis ↓protein synthesis signaling (mTOR/p70S6K/4E-BP1)	[58]
Hindlimb Suspension (Microgravity)	Sprague-Dawley rats (6 months, 28 days of HLS)	↓muscle mass	↑muscle atrophy ↑oxidative stress ↓antioxidant enzymes	[149]
	Fischer 344×Brown Norway inbred rats (34 months, 14 days of HLS)	↓muscle mass ↓muscle strength	↑autophagy ↑muscle atrophy (MuRF1) ↓satellite cell proliferation and differentiation	[150,151,152]

Animal models of sarcopenia with chronic diseases (e.g., cancer cachexia, chronic pulmonary diseases) are not included, we summarize animal models that have been widely used as in vivo models associated sarcopenia. ↑; increase, ↓; decrease, ↔; no change. Abbreviations: CSA; cross-sectional area, HFD; high-fat diet, HLS; hind-limb suspension.
